# Aberrant Stress Granule Dynamics and Aggrephagy in ALS Pathogenesis

**DOI:** 10.3390/cells10092247

**Published:** 2021-08-30

**Authors:** Yi Zhang, Jiayu Gu, Qiming Sun

**Affiliations:** 1 Department of Biochemistry, and Department of Cardiology of Second Affiliated Hospital, Zhejiang University School of Medicine, Hangzhou 310058, China; zhangyi_@zju.edu.cn (Y.Z.); joygu@zju.edu.cn (J.G.); 2 Chu Kochen Honors College, Zhejiang University, Hangzhou 310058, China; 3 Zhejiang Laboratory for Systems & Precision Medicine, Zhejiang University Medical Center, 1369 West Wenyi Road, Hangzhou 311121, China; 4 Zhejiang Provincial Key Laboratory of Genetic & Developmental Disorders, Hangzhou 310058, China

**Keywords:** stress granule, aggrephagy, neurodegenerative disease, amyotrophic lateral sclerosis, phase separation

## Abstract

Stress granules are conserved cytosolic ribonucleoprotein (RNP) compartments that undergo dynamic assembly and disassembly by phase separation in response to stressful conditions. Gene mutations may lead to aberrant phase separation of stress granules eliciting irreversible protein aggregations. A selective autophagy pathway called aggrephagy may partially alleviate the cytotoxicity mediated by these protein aggregates. Cells must perceive when and where the stress granules are transformed into toxic protein aggregates to initiate autophagosomal engulfment for subsequent autolysosomal degradation, therefore, maintaining cellular homeostasis. Indeed, defective aggrephagy has been causally linked to various neurodegenerative diseases, including amyotrophic lateral sclerosis (ALS). In this review, we discuss stress granules at the intersection of autophagy and ALS pathogenesis.

## 1. Introduction

Amyotrophic lateral sclerosis (ALS) is a fatal neurodegenerative disorder characterized by progressive degeneration of the upper and lower motor neurons, resulting in a loss of motor function and eventually death. About 10% of ALS cases are familial (fALS), while about 90% are sporadic (sALS). Identification of ALS-causative genes, including superoxide dismutase 1 (SOD1), transactive response DNA-binding protein 43 (TARDBP-43), fused in sarcoma (FUS), chromosome 9 open reading frame 72 (C9orf72), heterogeneous nuclear ribonucleoprotein A1 (HNRNPA1), valosin-containing protein (VCP), ubiquilin 2 (UBQLN2), sequestosome 1 (SQSTM1/p62), annexin A11 (ANXA11), optineurin (OPTN), and TANK (TRAF-associated NF-κB activator)-binding kinase 1 (TBK1) have advanced the understanding of ALS pathogenesis. ALS gene products are considered resident stress granule (SG) components or SG-associated proteins (Table 1) [1].

Stress granules are membrane-less organelles formed through the process of liquid–liquid phase separation (LLPS) under certain stress conditions, such as oxidative stress and heat shock, among others [1,2]. SGs are transient cellular compartments that undergo dynamic assembly and dissociation. However, chronic stress can lead to persistent stress granules, eventually resulting in the aggregation of disease-related proteins.

Macroautophagy (hereafter referred to as autophagy) is an evolutionarily conserved lysosomal degradative pathway, which is essential in the cellular and organismal levels of homeostasis [3,4,5]. Morphologically, autophagy is initiated by the formation of phagophores in mammalian cells. After nucleation of the phagophore, the membrane expands to generate an autophagosome, which fuses with a lysosome or vacuole, leading to the degradation of the cargo [6,7,8,9]. Clearance of the cytosolic components, such as protein aggregates, is conferred by cargo receptors that specifically recognize the cargo [10,11,12,13,14,15,16,17]. Dysfunction of autophagy is highly associated with various human diseases [18,19,20], such as neurodegenerative diseases.

**Figure 1:**
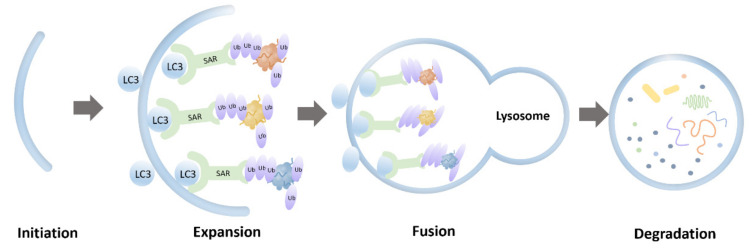
Schematic representation of aggrephagy.

Protein aggregates derived from interrupted SG dynamics pose a toxic insult, which can be partially mitigated by a selective autophagy pathway called aggrephagy (Figure 1). Aggresomes formed by those insoluble protein aggregates and labeled by ubiquitins are considered to initiate the process of aggrephagy. Aggresomes are transported to a microtubule-organizing center with the help of histone deacetylase 6 (HDAC6), which binds to ubiquitinated cargos [21]. Aggrephagy is controlled by a panel of receptor proteins, such as p62, next to BRCA1 gene 1 (NBR1), toll interacting protein (TOLLIP), OPTN, and Tax1 binding protein 1 (TAXBP1) [22]. Mechanistically, these receptors bridge ubiquitinated protein aggregates with autophagosomal membranes by simultaneously binding to ubiquitin chains and the lipidated LC3-family proteins via ubiquitin-associated (UBA) domains and LC3 interacting region (LIR) motifs, respectively [14,15]. These receptors are able to work both independently and cooperatively. For instance, NBR1 can interact with p62 and promote its phase separation [23]. Autophagy-linked FYVE-domain containing protein (ALFY) interacts with p62 and binds to several autophagy-related proteins, playing a role in the formation of autophagic membranes. The fusion between aggresomes and lysosomes involves proteins including Rab7, marking the final degradation of protein aggregates [21]. It should be noted that the mutations of two of these aggrephagy receptors, p62 and OPTN, are implicated in ALS.

Loss of SG homeostasis and defective aggrephagy are common pathological features of neurogenerative diseases [1,23,24,25,26,27,28]. VCP is encoded by an ALS causal gene and is a critical regulator mediating autophagic degradation of abnormal stress granules [29]. In this review, we will discuss the intersection of aggrephagy and stress granules in the pathogenesis of ALS.

## 2. Superoxide Dismutase 1 (SOD1)

Superoxide dismutase 1 gene encoding Cu/Zn superoxide dismutase was the first identified ALS-related gene [30]. The enzyme protects cells by detoxifying superoxide radicals O^2−^. SOD1 gene mutations account for approximately 20% of fALS. Although no consensus linking SOD1 mutations to toxicity has been reached [27], it is generally accepted that ubiquitinated cytoplasmic inclusions formed by ALS-causing SOD1 mutants contribute to toxicity in ALS [27]. Most ALS-associated mutations significantly impact the immature states of SOD1, destabilizing the metal-free and disulfide-reduced polypeptide, which leads to unfolding at physiological temperatures [31]. Moreover, mutations that change the hydrophobicity of SOD1 or cause cellular Ca^2+^ dysregulation promote the aggregation tendency of SOD1 mutants in ALS [32,33]. In addition, T cell-restricted intracellular antigen 1 (TIA-1) positive SGs can alter the dynamics of stress granules [34].

SOD1 is not a resident protein of SGs. However, mutant SOD1 interacts with TIA-1, one of the core components of stress granules associated with ALS. Mutant SOD1 increases the number of TIA-1 positive SGs. The abnormal interaction between mutant SOD1 and TIA-1 alters the dynamic of stress granules [34]. In addition, mutant SOD1 binds to GTPase-activating protein-(SH3 domain)-binding protein 1 (G3BP1), another protein marker of SGs, in an RNA-independent manner interfering with the dynamics of G3BP1-positive SGs [35]. Therefore, these findings suggest that aberrant interactions between SOD1, TIA-1, and G3BP1 might dysregulate SG.

Mutant SOD1 aggregates can be recognized by p62 and targeted for autophagic degradation [36,37]. Furthermore, mutant SOD1 aggregates may sequester OPTN, resulting in a reduced mitophagy flux, accounting for neurodegeneration [38]. However, whether the perturbation of SGs dynamics by SOD1 mutants impacts protein aggregation tendency remains unclear. Further studies are needed to confirm the exact role of aggrephagy in SOD1-associated ALS and the specific aggrephagy receptors involved [39].

## 3. Transactive Response DNA-Binding Protein 43 (TDP-43)

Transactive response DNA-binding protein 43 belongs to the heterogeneous ribonucleoprotein family. TDP-43 plays a critical role in diverse cellular processes, such as regulating RNA splicing, pre-microRNA processing, messenger RNA transport, and stress granule formation [40]. Hyper-phosphorylated, ubiquitinated, and cleaved TDP-43 aggregation has been identified as a pathological protein in disease-affected central nervous system regions [41]. Furthermore, TDP-43 has been detected as abnormal cytoplasmic aggregates in neurons and glia of more than 90% of ALS and 45% of frontotemporal dementia (FTD) cases [42].

TDP-43 can aggregate and propagate in a seed-dependent, self-templating, prion-like manner in vitro and in vivo [43]. Under chronic cell stress, TDP-43 is recruited to the cytoplasmic SGs, which evolve to form insoluble pathological aggregates [44,45]. TDP-43 also interacts with the four other ALS causal gene products, HNRNPA1, HNRNPA2B1, matrin 3 (MATR3), and UBQLN2 [46,47,48,49], which are resident proteins in SGs. The identification of TIA-1 as an ALS causal gene further reinforces the fact that TDP-43 in ALS is formed via altered LLPS [50]. These observations suggest that many ALS causal genes may converge on the TDP-43 pathway associated with pathologies.

Several studies have confirmed that autophagy plays a role in clearing TDP-43 aggregates. Significant colocalization between selective autophagy receptor p62 with TPD-43 aggregates was observed in ALS/FTD, indicating that the autophagy pathway could prevent the accumulation of TDP-43 aggregates [51]. In addition, VCP and OPTN appear to colocalize with TDP-43 inclusions in the spinal motor neurons of ALS patients [52]. Upregulation of autophagy leads to reduced TDP-43 proteinopathy in the nervous system of ALS/FTD transgenic mice models, which further validates the role of autophagy in mitigating toxicity of TDP-43 mutants [53,54]. Conversely, TDP-43 also plays a role in the regulation of autophagy by binding to ATG7 mRNA via RNA recognition motif 1(RRM1). Down-regulation of TDP-43 decreases ATG7 mRNA levels, which abolishes autophagosome expansion [55]. Furthermore, the loss of TDP-43 impairs the fusion of autophagosomes with lysosomes through decreasing dynactin 1, a component of the dynein-dynactin complex involved in lysosome transportation. The impaired fusion finally leads to the accumulation of immature autophagic vesicles blocking the autophagy-lysosome pathway [56].

## 4. Fused in Sarcoma (FUS)

FUS was first discovered in 1993 as a fusion oncogene in human liposarcoma located on chromosome 16 [57,58]. It contains 15 exons encoding a 526-amino acid protein. Moreover, it contains an *N*-terminal Gln-Gly-Ser-Tyr (QGSY)-rich domain, an RNA-recognition motif, three Arg-Gly-Gly repeat domains (RGG1-3), a zinc-finger motif and a C-terminal nuclear localization signal (NLS) [59]. In 2009, pathological inclusion bodies containing mutant FUS protein were recognized in fALS6 cases [60,61]. Approximately 2/3 of FUS mutations are located on exons 12–15, which encode zinc-finger motif, RGG2 and RGG3 domains, and the NLS. Other mutations are located on exons 3–6, encoding QGSY-rich and RGG1 domains. The C-terminal mutations are twice as likely to occur in fALS than in sALS, while mutations within exons 3–6 are more common in sALS. C-terminal ALS mutations are pathological, as they disrupt NLS [62,63]. They cause defective nuclear import of FUS and cytoplasmic mislocalization. Cytoplasmic FUS mislocalization leads to nuclear loss of function and triggers motor neuron death through a toxic gain of function [64].

Arginine residues in RGG motifs are required for phase separation of FUS. Loss of FUS arginine methylation promotes phase separation and SG association of FUS [65]. Prion-like domains of FUS are located on the QGSY-rich and C-terminal RGG2 domain, contributing to FUS phase separation and aggregation. ALS-associated FUS mutants can bind and sequester wild type (WT) FUS into cytoplasmic SGs [66], accelerating aberrant liquid to solid phase transition of stress granules [67]. The nuclear import receptor (NIR), also known as Transportin-1, recognizes the NLS domain; therefore, it chaperons FUS from the cytoplasm to the nucleus. NIRs can reverse aberrant phase separation and aggregation of proteins with prion-like domains, including FUS and TDP-43, to mitigate neurodegeneration in vivo [65,68].

R521C and P525L are two common FUS mutations associated with ALS. FUS-R521C causes DNA damage and RNA splicing defects [69]. It colocalizes with stress granules, significantly increasing SG assembly and persistence [70]. FUS-R521C-positive SGs were colocalized to LC3-positive autophagosomes accumulating in autophagy-deficient neurons, suggesting that autophagy is involved in the clearance of FUS mutants [71]. P525L FUS mutation causes early-onset of ALS [72]. P525L-positive SGs are more intense and larger than the WT. The PI3K/AKT/mTOR pathway inhibition increases autophagy by reducing FUS recruitment into SGs and reduces abnormal SGs linked to P525L FUS [73]. Accumulation of ubiquitinated proteins and autophagy receptor p62 was detected in neuronal cells with ALS-associated FUS mutation due to impaired autophagy [74]. However, overexpression of Rab1 rescued these defects, suggesting that Rab1 has a protective role in ALS [75].

## 5. Chromosome 9 Open Reading Frame 72 (C9ORF72)

The chromosome 9 open reading frame 72 gene consists of 11 exons with three main transcripts formed through a complex process of alternative splicing and produces two protein isoforms. It is found in almost 40% of familial ALS and FTD cases [76,77]. The GGGGCC hexanucleotide repeat expansion (HRE) in the first intron of C9orf72 is the most common genetic cause of both ALS and FTD. C9orf72 HRE-induced cytotoxicity has been demonstrated to be caused by loss- and gain-of-function mutations. The mutations lead to the loss of function of the C9orf72 protein. In contrast, sense or antisense RNAs generate pathogenic dipeptide repeat (DPR) aggregates, including poly-GA, poly-GP, poly-GR, poly-PA, and poly-PR [78,79], with a toxic gain of function.

DPR is commonly detected in p62-positive cytoplasmic inclusions, indicating that DPR is prone to aggregation [80]. Nucleocytoplasmic transport was defective, leading to an aberrant accumulation of SG-resident proteins within inclusions positive for TDP-43 in the C9orf72-HRE mice model, indicating that abnormal SGs response caused by C9orf72 may lead to TDP-43 proteinopathy in FTD/ALS [81]. More importantly, the early appearance of persistent pathological stress granules prior to significant pTDP-43 deposition implicates aberrant stress granule response as the key disease mechanism driving TDP-43 proteinopathy in C9orf72 FTD/ALS. Moreover, C9orf72 poly-GR aggregation enhances cytoplasmic TDP-43 aggregation and sequesters full-length TDP-43 through an RNA-independent mechanism, leading to TDP-43 proteinopathy in vivo [82,83].

C9orf72 HRE can also disrupt nucleocytoplasmic transport at the nuclear pore complex via hairpin and G-quadruplex secondary structures of RNA, which accounts for the nuclear depletion and cytoplasmic accumulation of RNA-binding proteins [84]. Knockdown or mutation of C9orf72 abolished SG formation, negatively impacted the expression of SG-associated proteins, including TIA-1 and HuR, and facilitated cell death [85]. Moreover, toxic arginine-rich dipeptide DPRs derived from C9orf72 HRE undergo LLPS and induce spontaneous stress granule assembly through eIF2α phosphorylation and G3BP1 [86]. Poly-GR can also impair stress granule dynamics by delaying SGs disassembly, preventing cells from generating effective stress response and consequently causing persistent cellular stress [87].

Moreover, accumulating evidence demonstrates that C9orf72 regulates different stages of the autophagy pathway as an essential activator [88]. C9orf72 forms a multiprotein complex with Smith–Magenis syndrome chromosomal region candidate gene 8 (SMCR8) and ATG101 [89,90]. The complex regulates the expression and activity of unc-51-like autophagy activating kinase 1 (ULK1), a key autophagy initiation factor [90]. A related study also showed that C9orf72 interacted with Rab1a and ULK1 to regulate Rab1a-dependent trafficking of the autophagy initiation complex to the phagophore [91]. Moreover, C9orf72 regulates autophagosome maturation by acting as the GEF of Rab7 [92]. A recent study also demonstrated that C9orf72 inhibited mTORC1 and activated TFEB, thereby globally activating the autophagy pathway. The above-mentioned autophagy activities of C9orf72 appear to be altered in ALS-associated C9orf72 mutations. However, it is unclear whether the protein aggregates of C9orf72 mutants are substrates of autophagy.

**Table 1:** The representative ALS-causative proteins.

Protein	Associated NDDs	Important Structures	Function	Main Pathogenesis	Role in SG Dynamics	References
TDP-43	FTD, ALS, PD, HD	C-terminal Glycine-rich domain, RNA recognition motifs (RRM1 and RRM2), nuclear localization signal and nuclear export signal	Regulates mRNA splicing, translation, transportation and stability	Mutations cause loss of TDP-43 nuclear function and cytoplasmic accumulation	SG component	Reviewed in [93]
FUS	FTD, ALS	*N* terminal prion-like domain, RNA recognition motif, C terminal nuclear localization signal	RNA- binding protein aids RNA transcription and splicing	Mutations on NLS impair the FUS nuclear transport causing cytoplasmic aggregation	SG component	Reviewed in [94]
C9ORF72	ALS, FTD	-	Affect transcription, translation and RNA transport	Abnormal hexanucleotide GGGGCC repeat amplification	Cause stress and interact with SG proteins	Reviewed in [95]
SOD1	ALS	-	An antioxidant enzyme detoxifying superoxide	Mutated SOD1 exposes hydrophobic surfaces and *N*-terminal short region increasing aggregation propensity	Cause stress and interact with SG proteins	Reviewed in [96]
UBQLN2	ALS, FTD	Ubiquitin-like domain (UBL), UBA, four stress-induced protein 1-like domains (STI-1 like), PXX domain	Directs misfolded or redundant proteins to the proteasome, acts in macroautophagy	Missense mutations	SG autophagic clearance	Reviewed in [97]
ANXA11	ALS	Four conserved annexin (ANX) domains, low-complexity domain (LCD)	Regulates cytokinesis, vesicle trafficking, apoptosis, intracellular Ca^2+^ homeostasis and stress granule dynamics	Missense mutations	Cause stress and interact with SG proteins	Reviewed in [98]
VCP	FTD, ALS	*N*-terminal domain, ATP-binding domains D1 and D2	DNA damage response, cell cycle control, autophagy, and SG clearance	Mutations disrupt the autophagic degradation of ubiquitinated proteins, resulting in the accumulation of non-degradative autophagosomes	SG component; SG autophagic clearance	Reviewed in [99]
MATR3	ALS, FTD, AD	Two tandem RNA-recognition motifs, two zinc finger domains	Alternative splicing, mRNA stability, transcription and mRNA nuclear export	Missense mutations	SG component	Reviewed in [100]

NDD: neurodegenerative disease; PD: Parkinson’s disease; AD: Alzheimer’s disease; HD: Huntington’s disease; ALS: amyotrophic lateral sclerosis; FTD: frontotemporal dementia.

## 6. p62

p62 was the first described autophagy receptor [101]. p62 is also named sequestosome 1 based on the ability to form aggregates [102]. Structurally, p62 contains various protein-binding domains, including an *N*-terminal Phox and Bem1 (PB1) domain, ZZ-type zinc finger domain, LIR, KEAP1 interacting region motifs (KIR), and UBA in the C-terminal. The formation of the phagosome and subsequent removal in selective autophagy is dependent on p62 [101]. Firstly, the UBA domain of p62 binds to ubiquitin chains of the target cargo, while the LIR motif interacts with ATG8 family proteins that are covalently attached to the inner membrane surface of the growing phagophore [11,103]. Thereafter, the oligomerization PB1 domain strengthens the interaction between the cargo and the phagophore. Finally, autophagosomes are completed and fuse with the lysosomes. It is important to point out that p62 undergoes LLPS upon association with polyubiquitinated substrates, and this process is vital for autophagy initiation and clearance of autophagy cargoes [104]. Therefore, it is not surprising that p62 is frequently observed in SGs and protein aggregates under different conditions.

Mutations in the p62 gene, which affects normal function, have been reported in ALS and FTD patients [105,106,107,108]. Notably, aggregation and phosphorylation of p62 were identified in a wide range of neurodegenerative diseases, including neurofibrillary tangles in AD [109], SOD1 aggregates in ALS [110], ubiquitin inclusions in PD, Lewy bodies in dementia [111], and huntingtin inclusion in HD [112]. Mutations of the PB1 domain have been reported in both ALS and FTD [113]. Further, deletion of the PB1 domain prevents p62 binding to mutant SOD1 [36]. P62 forms a complex with C9orf72 to recognize stress granules for degradation by autophagy, and a defect in this process is implicated in ALS pathogenesis [114]. Notably, TBK1 controls the autophagosomal engulfment of polyubiquitinated mitochondria through p62 phosphorylation. Unexpectedly, p62 overexpression was shown to significantly promote disease progression in the SOD1 H46R-expressing mice ALS model [115]. The causality of p62 in ALS is still ambiguous, despite evidence linking p62 to ALS.

## 7. Optineurin (OPTN)

Optineurin is an important autophagy receptor involved in autophagic clearance of damaged mitochondria [116] and protein aggregates [117]. OPTN consists of several domains that interact with different proteins, including multiple coiled-coil motifs, a basic leucine-zipper motif (bZIP), an LIR motif, a UBA, and a C-terminal zinc-finger domain [118,119]. It recognizes various protein aggregates via its C-terminal coiled-coil domain in a ubiquitin-independent manner.

OPTN depletion significantly increases protein aggregation in HeLa cells [120]. The UBA domain of OPTN colocalized with inclusion bodies formed by the truncated form of TDP43^ND251^ in Neuro2A cells. Overexpression of wild-type OPTN decreased inclusion bodies through K63-linked polyubiquitin-mediated autophagy. However, UBA mutants increased the accumulation of inclusion bodies [121]. OPTN facilitates LC3 lipidation and the expansion of the phagophore into the autophagosome by promoting the recruitment of the Atg12-5-16L1 complex to WIPI2-positive phagophores [122]. OPTN can also undergo post-translational modifications, such as ubiquitination and phosphorylation, affecting its functions and downstream signaling [119]. TBK1, another ALS-causal protein, phosphorylates OPTN to enhance interaction with LC3 and promote autophagy activities [117]. Moreover, OPTN is an important autophagy receptor in parkin-mediated mitophagy. The function of OPTN is disrupted by ALS-linked mutation [123], and phosphorylation of OPTN by TBK1 enhances its binding to Ub chains and promotes mitophagy [124]. TBK1 binds and phosphorylates both OPTN and p62, increasing their autophagy functions. Therefore, the three proteins are considered players in the causal pathway linking autophagy and ALS [125]

OPTN gene mutations have been identified in both fALS and sALS. Three types of OPTN mutations were discovered in fALS patients in 2010—a homozygous deletion of exon 5, a homozygous Q398X nonsense mutation, and a heterozygous E478G missense mutation within the UBA domain [126]. Later, more than 20 OPTN gene mutations were discovered [127]. The Q398X mutation causes a premature stop during translation, resulting in a deletion of the coiled coil 2 domain, which is necessary for binding to ubiquitin and the ubiquitinated receptor-interacting protein. The E478G mutation increased the immunoreactivity for OPTN in the cell body and the neurites. The increased amount and different distribution of the mutated protein might disturb neuronal functions and accelerate the formation of the inclusion body [126].

## 8. Future Perspectives

In this review, we summarize several proteins involved in the pathogenesis of ALS and aberrant SG dynamics from an aggrephagy perspective. Further studies are needed to explain why mutations of some genes only cause ALS, but not other neurogenerative diseases, while mutations of other ALS genes lead to different neuronal disorders. The link between OPTN, TBK1, and p62 is still not clear, and therefore, it is necessary to investigate mutations of these proteins that may be co-inherited, further promoting ALS. Most current studies focus on dysfunctional protein–protein interactions and phase separations. However, future studies need to focus on RNA alteration in the inclusion or stress granules to reveal the underlying mechanisms from other perspectives. More importantly, it remains poorly understood how autophagy discriminates healthy SGs, which can properly disassemble when the stress fades away, from the unhealthy SGs, which are irreversible and form toxic cytoplasmic inclusions. Further, the upstream signals and how they transduce to mobilize autophagy machinery for proper spatiotemporal control of the surveillance on protein aggregation in the context of ALS pathogenesis needs further exploration. In particular, future studies should reveal when and how aggrephagy receptors recognize specific protein aggregates and how they interact with different cargoes.

## Data Availability

Not applicable.
